# The incidence of atrial fibrillation, new oral anticoagulation, stroke, and significant bleeds in patients receiving a new dual-chamber pacemaker

**DOI:** 10.1016/j.ijcha.2023.101307

**Published:** 2023-11-18

**Authors:** Elias Parkkari, Ville Vanhala, Ronja Lindberg, Juho Tynkkynen, Jussi Hernesniemi

**Affiliations:** a Faculty of Medicine and Health Technology, Tampere University, Tampere, Finland; b Tays Hearth Hospital, Tampere University Hospital, Tampere, Finland; c Department of Radiology, Tampere University Hospital, Tampere, Finland; d Finnish Cardiovascular Research Center Tampere, Tampere, Finland

**Keywords:** Anticoagulation, Atrial fibrillation, Dual-chamber pacemaker, Remote monitoring, Stroke

## Abstract

**Background and objectives:**

Atrial fibrillation and flutter (AF/AFL) can be easily detected in patients who have a dual-chamber pacemaker (PM). This can result in a high detection rate of these arrhythmias especially if patients are monitored remotely and detection limits are sensitive.

**Materials and methods:**

A single-center retrospective registry analysis of 1,285 consecutive AF/AFL and anticoagulation naïve patients from a limited geographical area undergoing implantation of a new dual-chamber PM (between 2013 and 2019). Seven-year follow-up data for incident AF/AFL, initiation of new oral anticoagulation and for incident strokes and bleeds was obtained from an in-depth review of all relevant patient records including written medical records and death certificates detailing causes of death.

**Results:**

During the follow-up, mortality reached 22.2 % and cumulative incidence of AF/AFL, new anticoagulation, strokes, and bleeds were 52.6 %, 40.4 %, 4.7 % and 10.4 %. In 92.6 % of the cases, AF/AFL was discovered by PM. Remote monitoring was initiated in 67 % (n = 856). Risk factor adjusted mortality in this group was significantly lower when compared to patients in regular out-patient clinic controls (HR 0.45, 95 % CI 0.35–0.57). Despite of their better overall prognosis, the AF/AFL was discovered, and oral anticoagulation was initiated more often in remote monitoring group (HR 1.58, 95 % CI 1.23–1.79 for AF/AFL and HR 1.67, 95 % CI 1.33–2.09 for anticoagulation). There was no significant difference in the incidence of strokes or bleeds.

**Conclusions:**

The incidence of new AF/AFL is high in this population. Remote monitoring is associated with higher diagnostic yields of AF/AFL and initiated anticoagulation, but not with stroke and significant bleeds.

## 1 Introduction

Modern pacemakers (PM) can detect irregular rhythms such as atrial fibrillation (AF) and atrial flutter and can register the duration of these events. Modern PMs can be remotely monitored with online alert ability. Patients implanted with a dual-chamber pacemaker have a grown risk for various conditions and events. Previous smaller studies suggest that about 50 % of pacemaker patients have pacemaker-detected atrial fibrillation and a 1.2 % annual thromboembolic event (TE) rate [1], [2]. Healey and colleagues found that during a 2.5-year follow-up 34.7 % of the pacemaker patients enrolled had an atrial tachyarrhythmia (ATA) [3].

Patients with pacemaker-detected atrial fibrillation have a significantly increased risk for stroke or systemic embolism. According to the prospective, observational TRENDS study, pacemaker patients’ risk for TE is quantitatively linked to the AF burden. Having a high AF burden doubled the risk for TE and having a low burden seemed to have no effect on the TE risk. In a 1.4-year follow- up, patients with no AF or AF for <5.5 consecutive hours had an 1.1 % annual risk for TE. Patients with an AF episode longer than 5.5 h the same risk was 2.4 % [4]. In the prospective, observational ASSERT study the risk for stroke or systematic embolism with pacemaker patients with over 24 h AF episode, had an adjusted hazard ratio (HR) of 3.2 compared to patients without AF [5]. In a systematic review Sagris and others reported that the pacemaker patients with an atrial high-rate episode (AHRE) longer than 30 s had 4.4 times higher risk for the stroke than the patients without [6].

Remote monitoring of pacemakers has become more common once its benefits have been noticed. Remote monitoring reduces staff workload, evaluations, control checks and healthcare costs [7]. Remote monitoring doesn’t only save resources and time, but it can also become vitally important for the patient’s treatment as it is capable of early detection of atrial fibrillation which is one of the leading causes of stroke. In the prospective, non-interventional RAPID study the median evaluation delay for AHREs for the pacemaker patient group with remote monitoring on (RM-ON) was 79 days shorter than for the patient group with remote monitoring off (RM-OFF). In addition, therapy adjustments occurred 77 days earlier in the RM-ON group and there were 50 % less in-office visits compared to the RM-OFF group [8]. The randomized COMPAS trial showed similar results. RM-ON patients had 36 % less follow-ups compared to the control group during 18-month follow-up time. RM-ON group had a mean 117-day gain in the medical intervention delay compared to the control group. Moreover, RM-ON group experienced less major adverse events such as deaths, hospitalizations for complications related to the pacing system, and hospitalization for an adverse cardiovascular event [9]. In the SETAM randomized trial ATAs were detected not only earlier but also more frequently in the RM-ON group compared to the RM-OFF group. ATA was detected in 28 % of the RM-ON group patients and in 22 % of the RM-OFF group patients [10]. Early detection of AF speeds up the initiation of anticoagulation which can reduce the risk of stroke [7].

The goal of this retrospective study was to evaluate the incidence new atrial fibrillation of flutter, initiation of new oral anticoagulation and the incidence of stroke and significant bleeds among un-anticoagulated patients with no history of atrial fibrillation and implanted with a regular dual-chamber pacemaker (excluding all implantable cardioverter-defibrillators, cardiac resynchronization devices and bundle branch pacing devices). The incidence of these events was observed separately among RM-ON and RM-OFF patients. This study is based on all in-depth reviewed data of all patient records including also causes of death data and written accounts of the consequences and causes leading to death and thus presents the largest observational study of consecutive patients receiving a normal dual-chamber pacemaker with high-quality endpoint data for these events. We planned the follow-up to be much longer than in previous studies (mostly under three years) [6]. During the study period NOACs had replaced the VKA as the most prescribed anticoagulant, which also offers a new viewpoint in the anticoagulation treatment in patients with AHRE.

## 2 Methods

This study was based on a retrospective registry of consecutive patients undergoing dual-chamber pacemaker implantation in Tays Heart Hospital between 2013 and 2019. Tays Heart Hospital is the sole provider of specialized cardiologic care in the region of Pirkanmaa (geographical area with a catchment area of approximately 0.5 million inhabitants) and sole provider of cardiothoracic surgery services for a catchment area of over 1 million inhabitants. Between January 1st, 2013, and December 31st, 2019, 4,872 new pacemakers were implanted, and 1,690 old pacemakers were replaced. For the purpose of this study, we excluded patients residing outside the Pirkanmaa region (for lack of reliable follow-up data), patients implanted with an ICD, implanted with CRT device and patients implanted with single lead pacemaker and patients implanted with dual-chamber pacemaker but who already had oral anticoagulation therapy (for any indication) or who had previously been diagnosed with atrial fibrillation or flutter. After the exclusions, 1,285 patients were included in the final study population.

The study design was approved by the scientific monitoring board of Pirkanmaa hospital district. Due to the nature of the study, informed consent was not required. The study complies with the Declaration of Helsinki on the ethical principles for medical research.

### 2.1 Patient selection for remote monitoring and patient management

In Tays Heart Hospital, remote monitoring of dual-chamber pacemakers was only available in Boston Scientific devices. These devices and the included remote monitoring service was initially offered to patients with good overall prognosis and remote monitoring was usually not offered to patients with severe cognitive impairment and poor functional status. During the observation period, Boston scientific became the largest service provider of dual-chamber pacemakers.

In 2016 a decision was made to change the routine follow up protocol and to offer remote monitoring to every eligible patient who had a new pacemaker implantation. Patient was considered suitable for remote monitoring, if they were willing, were capable to use remote monitoring device or they had a care-taker capable to use remote monitoring device, and they weren’t distracted by the device. Attending physician made the decision to start the remote monitoring, usually during the implantation or in the first in-office follow up (usually three months after the implantation) by the attending physician. Remote monitoring device was given to the patient usually on the same day as the decision was made.

RM-ON patients had a routine in-office follow up every 4 years after the first in-office follow-up. Contact details of the pacemaker unit were given to the patients, and they were instructed to call if they had symptoms related to their heart disease or the pacemaker. Scheduled remote follow-ups weren’t routinely used, and patients needed to call the pacemaker unit to enable patient-initiated follow up. Only pre-defined alert events were used to detect arrhythmias or technical issues. If needed, in-office follow-ups and remote monitoring were tailored according to the patient’s needs.

RM-OFF patients had a routine in-office follow-up every 2 years after the first in-office follow-up at here months after implantation of the pacemaker. At signs of battery depletion, time interval between in-office follow-ups was decreased.

The individual group designations (RM-ON or RM-OFF) were fixed for each patient which means that during the follow-up, it was not generally changed.

Alerts episodes received from remote monitoring were revised by consulting cardiologists. Each AHRE-episode EGM strip was analyzed by the attending physician before the decision about anticoagulation therapy was made. The decision to initiate anticoagulation treatment was based on patients’ thromboembolic risk calculated by the CHA_2_DS_2_Vasc score (2 points ≥ anticoagulation indicated and 1 = anticoagulation considered based on individual risk and patient preferences) and estimated bleeding risk. During the observation period, pacemaker discovered asymptomatic AHR episodes lasting more than six minutes were usually considered significant enough to warrant evaluation for oral anticoagulation for protection against thromboembolic events.

### 2.2 Clinical phenotype data collection

Clinical phenotype data of preexisting conditions, medical treatments and operational details were collected from various sources and combined in a single dedicated study registry (MADDEC-study). The MADDEC-study focused on the prediction and detection of serious adverse events in cardiologic patients and the database comprises data from various sources: The KARDIO registry, electronic hospital records from specialized healthcare of the Pirkanmaa hospital district and all written records of all hospital visits [11]. The KARDIO-registry is a prospectively online updated structured database of all patients treated in Tampere Heart Hospital detailing patients pre-existing conditions and details of all invasive procedures and treatments during patient hospital stay. The hospital electronic health records comprise information of all laboratory and ECG measurements and hospital discharge diagnoses (in ICD-10 format) and diagnostic and interventional operations (in Nordic Medico-Statistical Committee classification of surgical and radiological procedures). Written electronic health records detail all events during patients’ hospital stays and emergency rooms visits.

### 2.3 Outcome definition, data collection and adjudication

Primary outcomes of this study were new diagnosis of atrial fibrillation or atrial flutter, newly initiated permanent anticoagulation in previously un-anticoagulated patient, significant bleeding event (BARC class ≥ 2 bleeding) and stroke diagnosed using the International Classification of Diseases-Tenth Revision (163 and 164) [12]. Stroke subtypes were classified based on written medical reports written by neurologists. The outcome data was collected by an in-depth review of all written patient records (including pacemaker monitoring reports) and written death certificate data detailing the consequences leading to and the final cause of death (also containing cause of death adjudication in ICD-10 format) and EHR data. For newly diagnosed atrial fibrillation or flutter, we further recorded how the diagnosis was made (discovery by pacemaker or by any health care provider) and the exact or estimated duration for the first AF/AFL episode.

### 2.4 Statistical analysis

The data is presented in means (and standard deviations) or by median (and interquartile range) and comparisons between continuous variables was performed by student’s *t*-test of by Kruskall Wallis depending on the distribution of the continuous variable. Comparisons across groups for categorical variable distributions was performed by Chi-square test (2-sided asymptomatic significance). The cumulative incidence of all primary outcome variables was determined by a survival analysis accounting for competing events by mortality during the follow-up using a sub-distribution hazard model but also limiting the follow-up times to seven years after which there was too few patients available for follow-up in the RM-ON group. This model was also used to compare the hazard ratios for incidence of primary outcome variables between RM-ON and RM-OFF patients. Hazard ratios are presented as unadjusted or adjusted for significant confounding variables selected to the final model by a p-value < 0.05 (selection by a backward stepwise algorithm). Additional analyses were also performed by including left atrial diameter measured from standard parasternal long axis images (data available in 65.9 %). Mortality in the entire study population was analyzed and comparison between different groups was performed by a Kaplan-Meier survival curve and by unadjusted and adjusted Cox-regression analysis. Conformation of the proportional hazard assumption for variables in survival analysis was verified by comparing correlation of survival times. Sensitivity analysis was also performed to verify the lack of significant interaction between study year and RM status associating with incidence of the main endpoints due to the increased proportion of patients in RM-ON group in later years of the observation period. A p-value ≤ 0.05 was considered significant. All analysis were by SPSS software (version 28.0) or by R software (packages survival and cmprisk).

## 3 Results

### 3.1 Demographics and overall mortality during the follow-up

The indication for implanting a pacemaker was most often sick sinus syndrome followed by disturbances in atrioventricular conduction with these covering a combined 88.7 % of all indications (Table 1.). Approximately two thirds of all patients were started on remote monitoring (66.56 %, n = 856) and their age distribution was significantly different when compared to patients attending regular out-patient clinic visits (median age 76 years with IQR of 68.25–81.00 vs median age 77 years with IQR 69–84, p < 0.0001). The proportion of patients with history of heart failure was smaller (12.8 % vs. 16.0 %, p = 0.015) and the proportion hypertensive patients was greater among patients with remote monitoring strategy (61.6 % vs 53.0 %, p = 0.003) when compared to patients in out-patient controls (Table 1.). Additionally, patients in RM-ON group had lower prevalence of dementia and slightly higher mean LVEF at baseline and lower ventricular pacing percentage at first control (Table 1.) Similarly, there were small but statistically significant differences in the distribution in indications between these two patient sets (Table 1.). The proportion of RM-ON patients increased significantly from the earliest years but reaching the highest proportion in 2017 and then plateauing: 9.4 % (2013), 32.4 % (2014), 62.6 % (2015), 83.4 % (2016), 88.0 % (2017), 81.0 % (2018), and 79.6 % (2019).

**Table 1:** Population characteristics of patients with no previous anticoagulation or history of atrial fibrillation undergoing dual-chamber pacemaker implantation in Tays Heart Hospital. Population is also stratified by the selected monitoring strategy.

	All n = 1285	Monitoring strategy*		
		RM-ON n = 856	RM-OFF n = 429	
Median Age (IQR)	76.0 (69.0–82.0)	76 (68.3–81.0)	77 (69.00–84.0)	<0.001
Men (n)	54.1 % (696)	55.7 % (477)	50.9 % (219)	0.104
Median Creatinine (IQR)	83.0 (70.0–100.0)	83.0 (69.8–99.9)	83.0 (70.3–100.0)	0.899
Coronary artery disease	21.0 % (267)	22.0 % (188)	19.1 % (79)	0.234
Previous MI	12.4 % (159)	12.9 % (110)	11.8 % (49)	0.603
Previous stroke or TIA	9.9 % (125)	9.5 % (81)	10.6 % (44)	0.522
Previous diabetes	23.7 % (302)	24.6 % (211)	21.7 % (91)	0.248
Heart failure**	12.8 % (165)	11.2 % (96)	16.0 % (96)	0.015
Cancer***	17.9 % (227)	17.8 % (152)	18.1 % (75)	0.883
COPD	4.5 % (57)	4.2 % (36)	5.1 % (21)	0.481
Dementia	8.1 % (103)	4.5 % (38)	15.6 % (65)	<0.001
Atrial pacing**** % (IQR)	22.0 % (4.0–50.0)	22.0 % (4.0–49.5)	22.0 % (4.0–54.0)	0.380
Ventricular pacing**** % (IQR)	50.0 % (2.0–99.0 %)	37.0 % (1.0–98.0)	74.5 % (5.0–99.0)	<0.001
Mean LVEF (SD)	58.5 % (9.6)	59.4 % (9.3)	56.7 % (10.0)	<0.001
Mean LA diameter (SD)	38.9 (6.4)	38.9 (6.6)	38.6 (5.9)	0.529
Hypertension	58.5 % (747)	61.6 % (527)	53.0 % (220)	0.003
Median CHA_2_DS_2_Vasc score	3 (2–4)	3 (2–4)	3 (2–4)	0.823
Indication				0.041
Sick Sinus Syndrome	31.5 % (405)	33.2 % (284)	28.1 % (121)	
1st degree AV block	2.2 % (28)	2.1 % (18)	2.3 % (10)	
2nd degree AV block	27.7 % (356)	28.0 % (240)	27.0 % (116)	
3rd degree AV block	29.5 % (379)	27.7 % (237)	33.0 % (142)	
Trifascicular block	6.5 % (83)	7.0 % (60)	5.3 % (23)	
Other	2.7 % (35)	2.0 % (17)	4.2 % (18)	

Abbreviations: IQR, Inter Quartile Range; MI, Myocardial Infarction; TIA, Transient Ischemic Attack; COPD, Chronic Obstructive Pulmonary Disease; LVEF, Left Ventricular Ejection Fraction; LA, Left Atrial.

* RM-ON denotes patients who were assigned to remote monitoring strategy, and RM-OFF denotes patients who attended regular in-office visits for pacemaker controls.

** History of decompensated heart failure or left ventricular ejection fraction < 35 %.

*** Any type of malignancy in remission or active.

**** Data available in 95.8 % for AP and VP, 65.9 % for LA diameter and 84.1 % for LVEF.

**Fig. 1:**
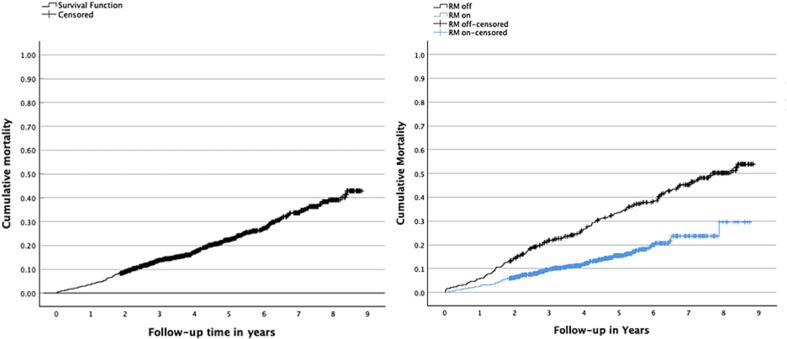
Overall mortality in patients undergoing pacemaker implantation and mortality stratified by remote monitoring status.

During the follow-up 300 patients died, of whom 40.0 % (n = 120) died from cardiac causes, 7.7 % (n = 23) died from other cardiovascular causes, and 52.3 % (n = 157) died from other causes. There was no statistically significant difference when comparing mortality to different causes in RM-ON and RM-OFF groups (p = 0.773). Median follow-up for overall mortality was 4.1 years (IQR 2.7–5.9) in the entire study population (3.9 years with IQR 2.7–5.2 in RM-ON group and 5.2 years with IQR 2.6–7.5 in the RM-OFF group). Cumulative mortality (by Kaplan Meier curves) in the entire study population and in both separate groups are presented in Fig. 1. As demonstrated by the mortality curves, the unadjusted mortality risk was significantly lower among RM-ON patients (HR 0.42 with 95 % CI 0.33–0.53, p < 0.0001). This association persisted even adjusting for all possible confounders indicating a significant residual confounding selection bias not covered by baseline demographics (HR 0.47 with 95 % CI 0.37–0.61, p < 0.001, analysis adjusted for all other risk factors presented in Table 1.).

### 3.2 Discovery of atrial fibrillation or flutter and initiation of anticoagulation during follow-up

**Fig. 2:**
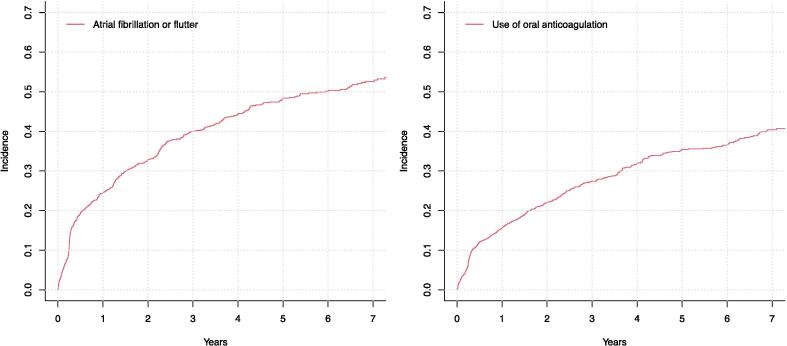
The cumulative incidence of new atrial fibrillation and flutter and initiation of oral anticoagulation in patients implanted with dual-chamber pacemaker.

The cumulative incidence of AF/AFL in the entire study population is presented in Fig. 2. Briefly, the overall cumulative incidence of AF/AFL reached 52.6 % at seven years (n = 583 events). Most often the discovery of atrial fibrillation was made on an episode discovered by the pacemaker system (92.6 % of the cases) and only in 7.4 % of the cases AF was discovered primarily by some other means than the pacemaker (either incidentally in other health care check-ups or when screening for AF/AFL due to symptoms) (Table 2). Most often the discovery was based on an episode lasting less than six minutes (31.3 %). Among RM-ON group, only 4.2 % (n = 17) of the AF/AFL discoveries were not made by the pacemaker, whereas the corresponding number was 14.6 % (n = 26) among RM-OFF group (p < 0.001). Corresponding to this, the duration of the first AF/ALF episode was usually also shorter in duration among RM-ON group (Table 2.).

**Table 2:** Duration of the first diagnosed atrial fibrillation (AF) or atrial flutter (AFL) episode.

	All n = 1285	Monitoring strategy		Sig**
		RM-ON	RM-OFF	
Duration of the first AF/AFL episode				0.006
<6 min	31.3 % (180)	33.3 % (133)	26.7 % (47)	
6–60 min	17.9 % (103)	19.5 % (78)	14.2 % (25)	
1–24 h	28.5 % (164)	28.7 % (115)	27.8 % (49)	
Over 24 h	8.3 % (48)	13.6 % (24)	6.0 % (24)	
Undetermined	14.1 % (81)	12.5 % (50)	17.6 % (31)	

*RM-ON denotes patients who were assigned to remote monitoring strategy, and RM-OFF denotes patients who attended regular in-office visits for pacemaker controls.

** Asymptomatic 2-sided difference in distribution by Chi square statistics.

**Fig. 3:**
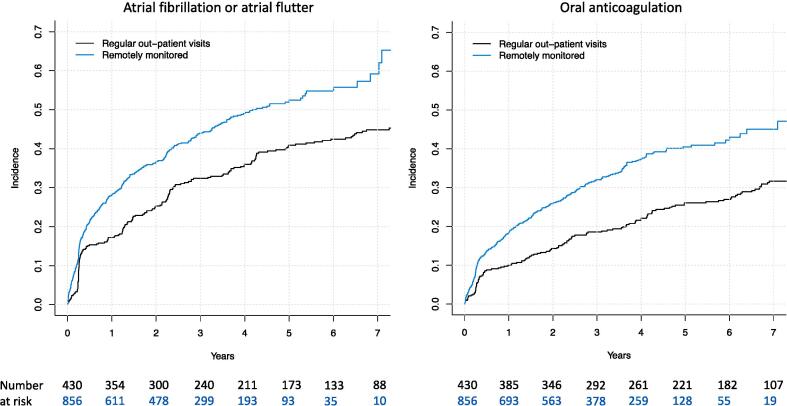
The cumulative incidence of new atrial fibrillation and flutter and initiation of oral anticoagulation in patients implanted with dual-chamber pacemaker stratified by pacemaker control strategy (p < 0.0001 for both comparisons).

There was a significant difference in the cumulative incidence of AF/AFL diagnoses among RM-ON and RM-OFF groups (59.2 % vs 44.8 % at seven years leading to a yearly incidence of 8.5 % and 6.4 %, p < 0.00001) (Fig. 3). The corresponding unadjusted and adjusted hazard ratios in the subdistribution hazard models were 1.52 (1.27–1.82) and 1.48 (1.23–1.79, model adjusted for age, sex, and prevalence of dementia, atrial pacing percentage at first three-month control). When the analysis was further adjusted for LA diameter (available in 65.9 %) the association remained significant and similar with a hazard ratio of 1.44 (1.13–1.84).

The cumulative incidence of newly started oral anticoagulation treatment reached 40.4 % in the entire study population (Fig. 2) (n = 431). As with AF/AFL diagnosis, the cumulative incidence of newly started oral anticoagulation treatment was significantly higher in the RM-ON group when compared to the RM-OFF group (45.0 % vs. 31.7 % at seven years leading to a yearly incidence of 6.4 % and 4.5 %, p < 0.00001). (Fig. 3.) The corresponding unadjusted and adjusted hazard ratios in the subdistribution hazard models were 1.71 (1.38–2.10) and 1.67 (1.33–2.09, model adjusted for age, sex, serum creatinine, prevalent dementia and atrial pacing percentage at first control). When the analysis was further adjusted for LA diameter, the association remained significant and similar with a hazard ratio of 1.61 (1.21–2.15). Most commonly the anticoagulation was initiated by a novel anticoagulant (76.2 %, n = 328). Warfarin was used in 23.8 % of the cases (n = 103).

### 3.3 The cumulative incidence of strokes and significant bleeding episodes

**Fig. 4:**
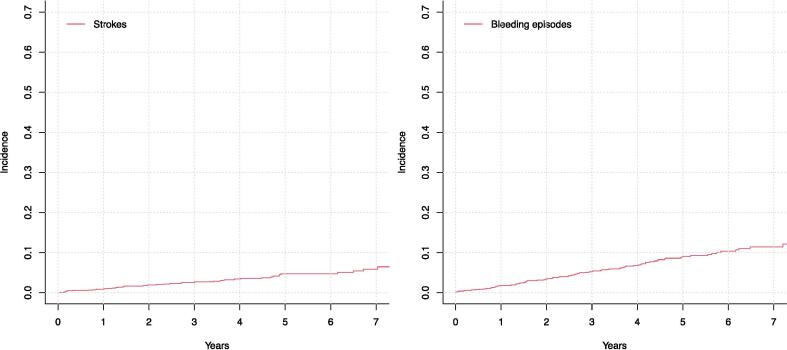
The cumulative incidence of strokes and significant bleeding episodes in patients implanted with dual-chamber pacemaker.

The cumulative incidence of strokes and significant bleeding episodes in the entire study population were 4.72 % and 10.4 % at seven years (Fig. 4). Of all strokes (n = 48), 85.1 % were ischemic and 14.9 % (n = 7) were caused by intracranial hemorrhage. Stroke was fatal in 34.0 % (n = 16) of the cases with intracranial hemorrhages accounting for a disproportionate amount of the stroke deaths (37.5 %, n = 6/16). As for the bleeding events (total of n = 94), 46.8 % (n = 44) were not severe (BARC class 2), 43.6 % were classified as severe BARC 3a/b bleeds and 9.6 % as severe BARC class 5 bleeds (intracranial bleeds). The bleeding event resulted in death in 13.8 % of the cases (n = 13, of which 6 were hemorrhagic strokes as presented above). In 52.1 % (n = 49/94) of the bleeding events, the patient had been previously started on anticoagulative therapy. But overall, in patients who had been previously started on anticoagulation the bleeding events were less often severe (BARC classes 2) when compared to patients who had not been started on anticoagulation (57.1 % [n = 28] vs 35.6 % [n = 16], p = 0.036).

**Fig. 5:**
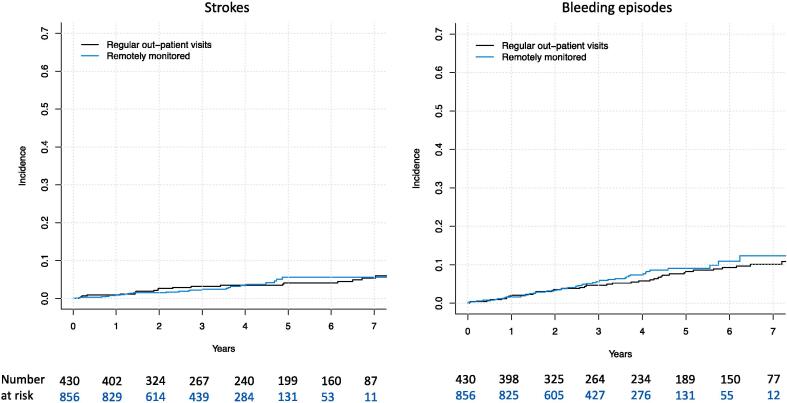
The cumulative incidence of strokes and significant bleeding episodes in patients implanted with dual-chamber pacemaker stratified by pacemaker monitoring strategy (p = ns for both comparisons).

Despite the great difference in the cumulative incidence of AF/AFL and started oral anticoagulation therapy, there was no significant difference in the cumulative incidence of strokes and significant bleeding episodes in different monitoring strategies (5.6 % vs 4.1 % at seven years leading to a yearly incidence of 0.80 % and 0.58 %, [p = 0.811] for strokes and 10.9 % vs 9.3 % at seven years leading to a yearly incidence of 1.6 % and 1.3 %, [p = 0.463] for significant bleeding episodes when comparing RM-ON and RM-OFF groups) (Fig. 5.). The corresponding unadjusted and adjusted hazard ratios in the subdistribution hazard models for stroke were 0.99 (0.54–1.91) and 1.10 (0.31–0.32, model adjusted for age, diabetes, prevalent cancer and serum creatinine) and 1.09 (0.72–1.65) and 1.07 (0.70–1.63, model adjusted for age, prevalent hypertension and presence of sick sinus) for bleeding events.

The distribution of patients in above mentioned three bleeding classes was similar regardless of the originally selected pacemaker monitoring strategy (not severe BARC 2 bleeds 48.2 % [n = 27] vs 44.7 % [n = 17], severe BARC 3 class bleeds 42.9 % [n = 24] vs 44.7 % [n = 17] and severe BARC 5 bleeds 8.9 % [n = 5] vs 10.5 % [n = 4], when comparing RM-ON and RM-OFF groups, p = 0.934). Also, there was no significant difference in the proportion of fatal bleeds between these two groups (10.6 % [n = 4] vs 18.4 % [n = 7], p = 0.288) although the small number of events results in unpowered statistical comparison.

## 4 Discussion

In this retrospective registry study, we followed 1285 atrial fibrillation naïve patients (no anticoagulation at baseline) after the implantation of a normal dual-chamber pacemaker for a maximum of seven years*.* Overall, 67 % of the patients were remotely monitored. Overall mortality among these patients was 22 % and half of the patients developed atrial fibrillation. The vast majority (>90 %) of new-onset atrial fibrillation episodes were discovered by the pacemaker. Patients in RM-ON group were observed to have > 50 % lower overall mortality when compared to RM-OFF group. However, atrial fibrillation was discovered, and anticoagulation was initiated more often in the RM-ON group. There was no significant difference in the cumulative incidence of strokes or significant bleeds between these two groups.

Despite the very significant difference in overall mortality between the two groups, RM-ON and RM-OFF groups had only relatively small, although statistically significant, differences baseline age, prevalence of heart failure and hypertension, and in the indication of pacemaker implantation. In practice, the difference in overall prognosis between these groups is most likely explained by the fact that, especially in the beginning of the observation period, patients who had cognitive problem, poor compliance and/or clinically estimated poor overall prognosis were not usually offered the remote monitoring option. In short, a significant selection bias is likely to explain many of the observed differences between these two groups. The increase in the proportion of the patients in RM-ON group increased significantly during the follow up, from 9 % in 2013 to over 80 % in 2016–2019 due to changes in the hospital’s clinical policy.

Most of the incident AF/AFL were discovered by the pacemaker (93 %). Cumulative incidence of new AF/AFL was 53 % at seven years which is higher than the 10 % in the ASSERT study with a mean follow-up time of 2.5 years. The corresponding incidence between years 2 and 3 in our study were 33–40 % (data not shown). In the ASSERT study only atrial high rhythm episodes of over 6 min were considered as AF/AFL which might explain some of the difference [3], [5]. In our registry 31.3 % of all first observed episodes of AF/AFL had a duration below 6 min but since the cumulative incidence of initiation of anticoagulation reached 40 %, it is clear that at least that many patients had AF/AFL episodes of over 6 min which was considered the absolute minimum duration to warrant anticoagulation in our center. Previously, the Veterans Health Administration study has described anticoagulation prescription variation in patients, who had a new device-detected AF. During mean follow up of three years, 45 % of patients had AHRE > 6 min [13]. Similarly, in The Loop Study, which randomized 70–90 years old patients without atrial fibrillation and at least one CHA2DS2VASc-score besides age to receive implantable loop recorder (ILR) or usual care, 32 % of the patients in the ILR-group had atrial fibrillation of at least 6 min during a follow-up of little over 5 years [14].

According to our observations, RM-ON patients had higher incidence of AF/AFL (59 % vs. 45 %), even though there wasn’t any substantial difference in patients median age or CHA_2_DS_2_VASc-score. Proportion of the patients with first AF/AFL > 6 min was almost the same in both groups (31 % vs. 30 %). Our data is derived from the written records of hospital visits detailing the observations made in the interrogation of the pacemaker and in home monitoring alerts, for this reason it is unlikely that physicians in our institution could have been more prone (or sensitive) to make the AF/AFL-diagnosis in the RM-ON group when compared to RM-OFF group. However, the time-to-diagnosis could very likely be prolonged in the RM-OFF group because, in majority of cases the interval between out-patient control visits was two years. Furthermore, we didn’t collect any data about AF burden (percent of time within previous year or total time in AF/AFL in previous year) calculated by the pacemaker prior to AF/AFL-diagnosis, which may have affected clinical decision making.

During the study period (2013–2019), the clinical significance of AHRE was under debate. EHRA consensus statement (2017) recommended anticoagulation in patients with AHRE lasting longer than 5,5 h, if patient had CHA_2_DS_2_VASc > 2 (without 1 point for females). The consensus statement also acknowledged that mere 5-minute AHRE could increase the risk of stroke [15]. AHA/ACC/HRS Focused Update of the 2014 AHA/ACC/HRS Guideline for the Management of Patients with Atrial Fibrillation (2019) suggested that duration of AHRE, patients stroke risk and bleeding risk should be evaluated before anticoagulation therapy [16]. It should be also noted that risk of ischemic stroke, systemic arterial embolization or transient ischemic attack in patients with/without AHRE is increased as CHA_2_DS_2_VASc score is increased [17]. Clinical practice in our hospital was to calculate CHA_2_DS_2_VASc score, check recent laboratory results and medical history, assess bleeding risk, and then make a decision about anticoagulation therapy.

In our study, the cumulative incidence of initiated anticoagulation was 40 % (45 % of the patients in RM-ON and 32 % in the RM-OFF-groups). This cumulative incidence is smaller than the cumulative incidence of AF/AFL (52.6 %), but the difference is likely to be explained by the fact that in many patients the duration of AF/AFL episodes were under 6 min and in some patient the bleeding risk might have been estimated too high to initiate anticoagulation. The difference between RM-ON and RM-OFF groups (71 % higher chance for anticoagulation initiation in the RM-ON group) is likely to be explained by the higher cumulative incidence of AF/AFL in the RM-ON group (58 % higher chance of AF/AFL) and possibly due to *a priori* perceived higher bleeding risks or lack of utility of anticoagulation among the participants of the RM-OFF group. Unfortunately, we lack the data to make that conclusion.

Cumulative incidence of ischemic stroke was 3.2 %, and 10.4 % of patients experienced a bleeding event, of which 53 % were severe (BARC class ≥ 3). Fortunately, anticoagulation therapy wasn’t associated with poorer prognosis in a case of bleeding event. Even though patients in RM-ON had higher cumulative incidence for the initiation of anticoagulation therapy, PM monitoring strategy was not associated with patients’ risk of stroke or bleeding events. In the randomized and controlled LOOP Study, patients randomized to receive an implantable loop recorder (ILR-group) anticoagulation was more frequently initiated than in the control group (29.7 % vs. 13.1 % in control group), but this didn’t reduce the risk of ischemic stroke, systemic arterial embolization, or transient ischemic attacks (6.4 % vs. 7.0 %). In the LOOP study, the risk of thromboembolic events was higher than in our study, reflecting patients higher CHA_2_DS_2_VASc score (median 4 points in the LOOP Study, median 3 points in our study). There was no statistically significant difference in bleeding events between groups in the LOOP study (4.3 % vs 3.5 %) [14] which corresponds with our observations.

Recently published NOAF AFNET 6 trial compared edoxaban to placebo in AF and anticoagulation naïve patients with device detected atrial fibrillation lasting > 6 min. The trial was stopped early, after median follow-up of 21 months, because of safety and futility issues (primary endpoint of stroke, systemic embolism or cardiovascular: 3.2 % vs 4.0 % per patient year, p = NS). Anticoagulation led to a higher incidence of major bleeding (2.1 % vs. 1.0 % per patient years, p = 0.002). It’s noteworthy that 18 % of the patients had an ECG-based AF diagnosis during the trial. NOAH AFNET study had lower than expected stroke risk on a placebo group (1.1 % per patient year), despite the high median CHA2DS2VASc score (4) [18]. NOAH AFNET trial result is in line with the LOOP study and our study, showing that starting anticoagulation in patients with short AHRE episodes/subclinical AF is futile. Interestingly The LOOP study and our study didn’t demonstrate any bleeding risk with increased prescription rate of anticoagulants [14]. This difference might be explained by the differences in anticoagulants used or in study populations.

In the prospective ASSERT study, which enrolled AF/AFL naïve patients implanted with a new pacemaker (and no anticoagulation), risk of ischemic stroke or systemic arterial embolization was 4.2 % if patient had at least one AHR-episode, and the risk was 1.7 % if patient hadn’t experienced an AHR-episodes. Mean CHA_2_DS_2_VASc score was 2.2 in patients with AHRE and 2.3 in patients without AHRE [3]. In a previous retrospective analysis of remotely monitored patients implanted most often with implantable cardioverter-defibrillator and who participated the Veterans Health Administration study, incidence of stroke was 3.4 % in patients with mean CHA_2_DS_2_VASc score of 3.9 (median follow-up approximately 3.8 years). Bleeding risk wasn’t reported, but the prescription of OAC was associated with lower risk stroke, if AHRE lasted longer than 24 h [13].

In contrast to many previous studies with data of PM remote monitoring or the incidence of serious adverse events, our observations are based on a population with normal dual-chamber PMs (all ICDs and CRT-devices excluded). Additionally, the research center is the only provider of specialized care (cardiologic, neurologic, surgical, emergency medicine etc.) in the region, leading to reliable follow-up data with no losses to follow-up. Mortality statistics with causes of deaths were received from national registry (Statistics Finland) which records the cause of deaths for all Finnish citizens and people permanently residing in Finland (no-loss to follow-up and with high reliability) [19].

## 5 Conclusion

In this retrospective observational study, we found that the incidence of AF/AFL and the initiation of anticoagulation therapy is common (53 % and 40 %) in a long 7 year follow up of AF naïve patients with *de novo* implanted dual-chamber pacemakers. Remote monitoring is associated with higher diagnostic yields of AF/AFL and anticoagulation therapy, but this observation might be affected by significant selection bias. Remote monitoring strategy, although associated with increased cumulative incidence of initiated anticoagulation therapy, wasn’t associated with patients’ risk of ischemic stroke or risk of significant bleeding events. Which patients with AHRE benefit from anticoagulation is still unknown, but our results provide much needed data on the event rates of stroke and significant and severe bleeding events in this population. Hopefully, on-going ARTESiA trial will provide much needed data in the future on the utility of anticoagulation in device detected AF/AFL [20]. Current evidence suggests that the presence of AHRE (>6 min) should act as a warning signal for the physician to search for clinical AF, rather than an indication of an anticoagulation.

## Funding

This study was funded by the Terttu Vattula Testament Foundation.

## Declaration of competing interest

The authors declare that they have no known competing financial interests or personal relationships that could have appeared to influence the work reported in this paper.
